# NTRK-rearranged spindle cell sarcoma of the uterine cervix with a novel NUMA1::NTRK1 fusion

**DOI:** 10.1007/s00428-023-03724-1

**Published:** 2023-12-28

**Authors:** Luca Szalai, Ildikó Vereczkey, Marianna Szemes, András Rókusz, Erzsébet Csernák, Erika Tóth, Zsombor Melegh

**Affiliations:** 1 Department of Surgical and Molecular Pathology, National Tumour Biology Laboratory, National Institute of Oncology, Budapest, Hungary; 2 Bristol, UK; 3 Department of Pathology and Experimental Cancer Research, Semmelweis University, Budapest, Hungary

**Keywords:** NTRK fusion, NUMA1::NTRK1, Uterine sarcoma, Uterine cervix

## Abstract

*NTRK*-rearranged uterine sarcoma is a recently described entity that represents a subset of uterine sarcomas with distinct clinicopathological features. From a molecular point of view, this tumour is defined by *NTRK* gene rearrangement, resulting in overexpression or constitutive activation of Trk receptors. The presence of *NTRK* fusion is indicative of treatment response with a selective small-molecule inhibitor of the Trk kinases. Here, we report a case of an *NTRK*-rearranged sarcoma of the uterine cervix in a 43-year-old patient, measuring 80 mm in its largest dimension, with a novel *NUMA1*-*NTRK1* fusion, not previously reported in *NTRK*-rearranged uterine sarcomas or other *NTRK*-rearranged tumours. The fusion, involving *NUMA1* exon 14 (NM_006185.4) and *NTRK1* exon 11 (NM_002529.4), was identified by next-generation sequencing (NGS) studies (FusionPlex Pan Solid Tumor v2 panel). Although the presence of *NTRK* fusion has been reported in a variety of neoplasms, a fusion involving *NUMA1* (nuclear mitotic apparatus protein 1) and a tyrosine kinase partner has previously been reported in human neoplasms only in a handful of cases. The resulting fusion protein comprises the oligomerization domain of NUMA1, which is predicted to cause constant activation of the tyrosine kinase domain of NTRK1. The recognition and accurate diagnosis of these tumours are important due to the availability of potential targeted therapeutic options.

## Introduction

In recent years, several new, molecularly defined subtypes of uterine sarcomas have been described, which may have previously been diagnosed as fibrosarcoma, adenosarcoma or neurofibrosarcoma/fibroblastic peripheral nerve sheath tumour [1]. One of these newly defined entities is *NTRK*-rearranged uterine sarcoma. This tumour, which has a fibrosarcoma-like morphology and propensity to the uterine cervix, was first described by Mills et al. [1] and later characterised as an *NTRK*-rearranged sarcoma by Chiang et al. in 2018 [2]. These tumours generally consist of the proliferation of relatively monomorphic spindle cells with only mild to moderate nuclear atypia. Despite their morphologically bland appearance and often organ-confined nature at the first diagnosis, these tumours show a propensity for an aggressive clinical course with rapid recurrence and metastatic spread. Based on the relevant literature, less than 50 *NTRK*-rearranged uterine sarcomas have been described thus far, and various *NTRK* fusion partners have been identified [3–7]. In this study, we present an additional *NTRK*-rearranged uterine sarcoma with a novel *NTRK* fusion partner, which, to the best of our knowledge, has not been described to date.

## Case presentation

### Clinical findings

A 43-year-old female patient presented with irregular vaginal bleeding. CT imaging revealed a lesion infiltrating the uterine cervix and extending into the lower uterine segment. The lesion had a maximum diameter of 80 mm. After an initial core biopsy sampling, which raised the possibility of an *NTRK*-rearranged uterine sarcoma, the patient underwent a hysterectomy and bilateral adnexectomy.

### Gross examination

**Fig. 1:**
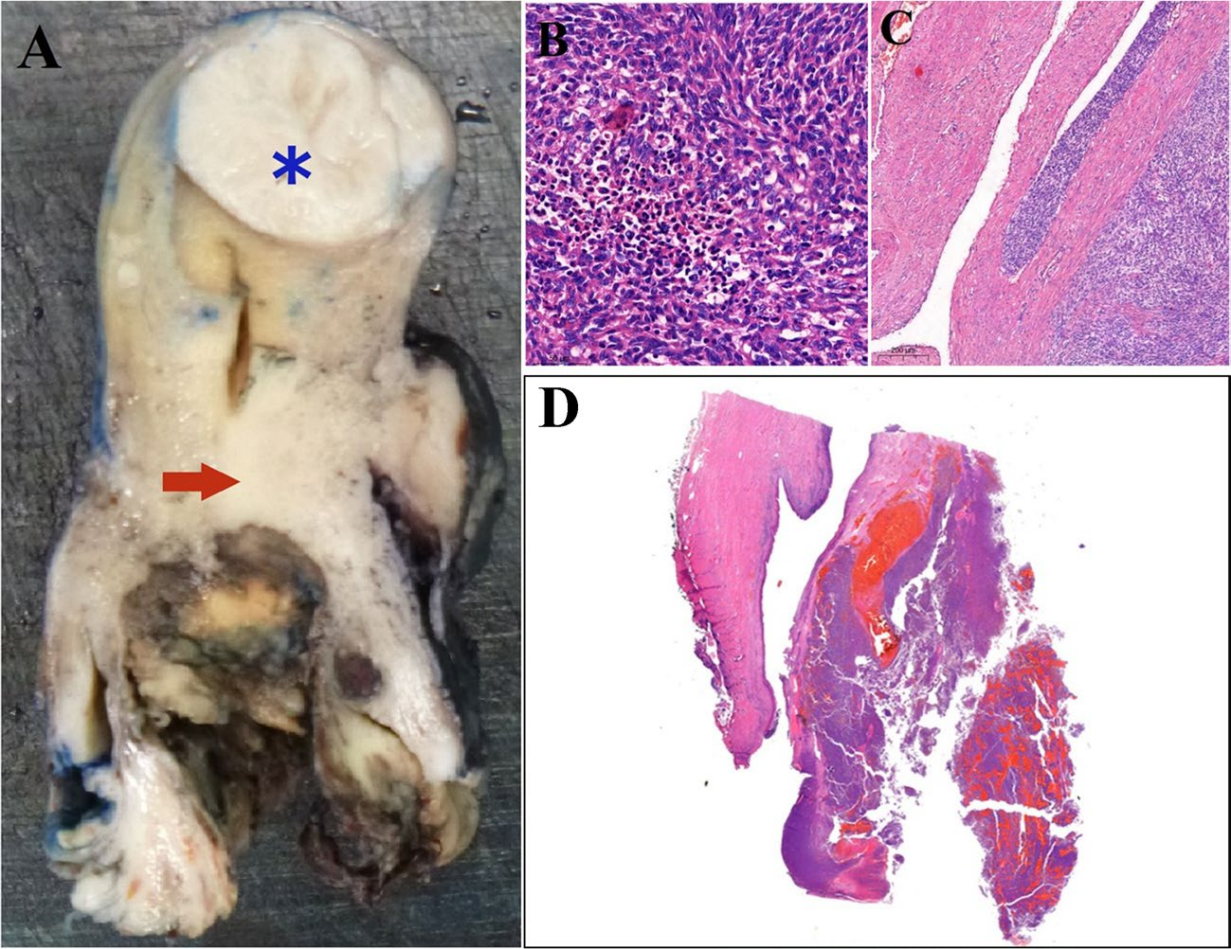
Macroscopically, the lesion was located in the cervix and had a greyish-white polypoid appearance with indistinct borders (**A**, **D)**. The lesion is indicated by a red arrow. A leiomyoma, which is marked by a blue star, could also be identified in the myometrium (**A**). Microscopically, neoplastic spindle cells arranged haphazardly and in a streaming pattern. Moderate lymphocytic infiltration between the tumour cells (**B**). Lymphovascular invasion can be seen (**C**) (HE, × 200 and × 100 magnification)

Macroscopically, a greyish-white, partly endophytic, polypoid lesion with a maximum diameter of 80 mm and indistinct borders filled the entire posterior cervix (Fig. 1A). The lesion macroscopically appeared confined to the cervix and did not clearly infiltrate the lower uterine segment. Retained cervical mucosa was not identified macroscopically. In the background myometrium, multiple leiomyomata were identified, measuring between 2 and 33 mm.

### Histology and immunohistochemical study

Histology of both the biopsy and the resection specimens revealed a highly cellular tumour, comprising relatively monomorphic spindle cells with bland, uniform nuclei, clumped chromatin and inconspicuous nucleoli. The tumour cells had a narrow eosinophilic cytoplasm and elongated nuclei. The tumour cells were arranged haphazardly or in a streaming pattern and infiltrated the cervix with a predominantly pushing border (Fig. 1B, D). There was extensive haemorrhage and necrosis within the lesion. There was a high mitotic activity with a mitotic count of 26 mitoses per 10 high-power fields. Lymphovascular invasion was also identified (Fig. 1C). A moderate infiltrate of mostly lymphocytes and plasma cells was also observed throughout the lesion.

**Fig. 2:**
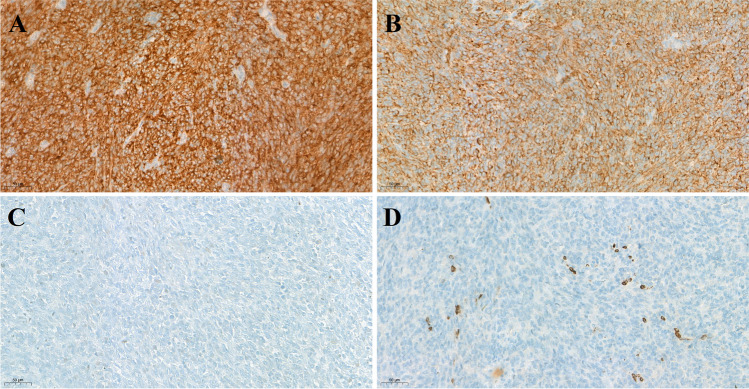
Neoplastic spindle cells demonstrating strong and diffuse membranous pan-Trk (**A**) and CD34 (**B**) positivity. The tumour cells are negative for S100 (**C**), and a small number of tumour cells are positive for desmin (**D**) (× 200 magnification)

Immunohistochemistry performed on both the biopsy and hysterectomy specimens demonstrated strong and diffuse membranous pan-Trk positivity (Fig. 2A). The tumour cells were also positive for CD34 but negative for S100 (Fig. 2B, C). There was focal positivity with cyclin D1 and CD10. A small number of tumour cells were positive for desmin and SMA (Fig. 2D). The tumor cells showed variable positivity with p53, which was considered a wild-type staining phenotype. The tumour cells were negative for ER, PR, p16 and h-caldesmon.

### Molecular analysis

Potential translocations were assessed by next-generation sequencing (NGS) using the FusionPlex Pan Solid Tumor v2 panel, which targets key fusions and variants relevant across solid tumours and sarcomas in 137 genes. Sequencing was performed on RNA obtained from the biopsy. The NGS panel revealed the presence of a novel fusion involving *NUMA1* exon 14 (NM_006185.4) and *NTRK1* exon 11 (NM_002529.4) with breakpoints located at chr11:71,727,454 and chr1:156,844,698 (GRCh37/hg19). The translocation preserved the reading frame, producing a fusion protein.

### Follow-up

Two months after the surgery, the patient developed a recurrence of right pelvic and retroperitoneal lymphadenopathy and secondary right iliac vein compression and thrombosis. She received three courses of adriamycin therapy and radiotherapy. Despite treatment, the tumour showed further progression. Seven months after surgery, she is currently being considered for immunotherapy and selective tyrosine kinase inhibitor (entrectinib) therapy.

## Discussion

*NTRK*-rearranged uterine sarcoma was first described by Mills et al. as a fibroblastic malignant peripheral nerve sheath tumor (neurofibrosarcoma) in 2011 [1] and later characterised as an *NTRK*-rearranged sarcoma by Chiang et al. in 2018 [2]. It represents a subset of uterine sarcomas with distinct clinicopathological features. The tumour occurs mainly in premenopausal women. The most common presentation is abnormal vaginal bleeding. The tumours are usually located in the uterine cervix and less often in the uterine corpus [8, 9]. Macroscopically, the tumour is usually poorly circumscribed or rarely polypoid with a yellow, pink or white cut surface [5, 8]. The majority of the lesions are FIGO stage IA or IB at the time of presentation [3, 5]. Microscopically, the tumours have a fibrosarcoma-like morphology with relatively uniform spindle cells and scant cytoplasm. Nuclear atypia is usually mild to moderate. The mitotic activity varies highly between tumours. The tumour cells are typically arranged haphazardly, but a fascicular growth pattern can also be seen. Most of the tumours have an infiltrative or occasionally a pushing border. The presence of a lymphoid infiltrate within the lesion is also common. Perivascular hyalinization and necrosis are present in approximately half of cases [5, 8]. On immunohistochemistry, the tumour cells demonstrate pan-Trk-positive staining in the vast majority of the tumours. Pan-Trk immunoreactivity is associated with a fusion partner-specific pattern of staining [2] *NTRK*-rearranged tumours also commonly express CD34, often with coexpression of S100, but lack SOX10 expression [4]. Our lesion showed strong membranous pan-Trk staining and CD34 positivity, but S100 was negative (Fig. 2A–C). The tumours tend to lack smooth muscle differentiation and are usually negative for desmin and h-caldesmon but may show focal positivity for SMA. While this tumour was h-caldesmon negative, a small number of tumour cells were positive for SMA and desmin (Fig. 2D). Hormone receptors are usually negative [6, 8].

In a recent study, the presence of lymphovascular invasion, necrosis, mitotic count ≥ 8/10 HPF and *NTRK3* fusion were considered poor prognostic factors in *NTRK*-rearranged uterine sarcomas. Tumours without any of these characteristics can be classified as low risk, while tumours with one or more of these characteristics can be considered high risk [3]. Our lesion fulfilled all but one of these criteria; thus, it was considered high risk.

**Fig. 3:**
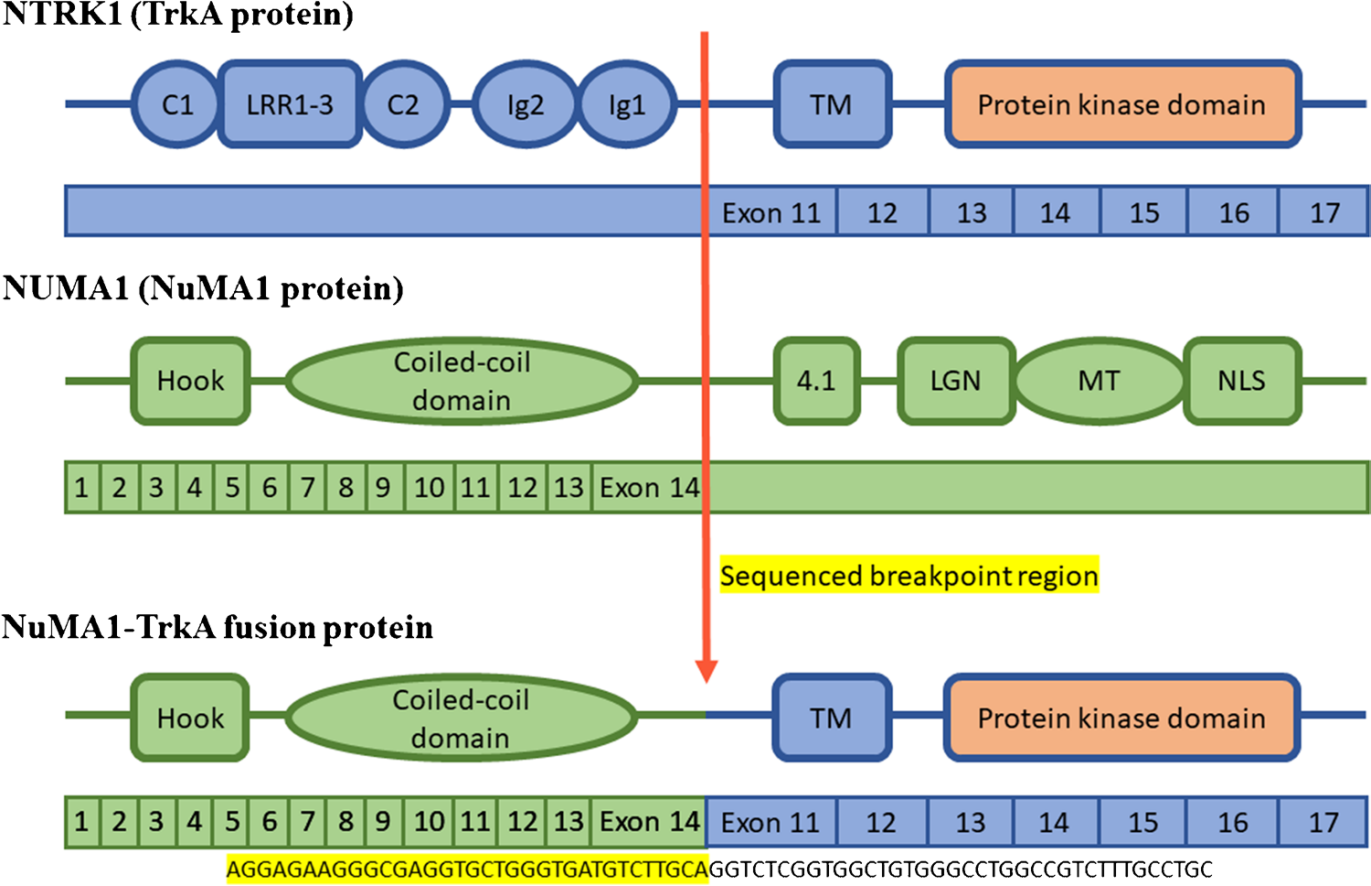
NuMA1-TrkA fusion protein. C1 and C2, cysteine-rich cluster; LRR1-3, three leucine-rich 24-residue repeats; Ig1 and Ig2, immunoglobulin-like domains; TM, transmembrane domain; 4.1, 4.1 family protein domain; LGN, LGN binding domain; MT, microtubule binding domain; NLS, nuclear localization sequence domain

The presence of *NTRK* fusions has been reported in a variety of tumour types with different morphologies and aetiologies. The *NTRK* genes—*NTRK1*, *NTRK2* and *NTRK3*—encode members of the tropomyosin receptor kinase (Trk) family, which include three transmembrane protein receptors TrkA, TrkB and TrkC. These are primarily involved in neuronal development and maintenance. Gene fusions involving *NTRK* genes lead to constitutive activation or overexpression of Trk receptors, promoting oncogenesis by activating the PI3K and MAPK pathways [10]. The significance of these genetic alterations lies in the new therapeutic opportunities they offer. Larotrectinib and entrectinib are first-generation *NTRK* inhibitors that have demonstrated a 57% therapeutic response in solid adult tumours harbouring *NTRK* fusions [8]. In *NTRK*-rearranged uterine sarcomas, *NTRK1* is more frequently involved in gene fusions than *NTRK3* [3], and *TPM3::NTRK1* fusion is the most common translocation reported [4]. Other reported *NTRK1* fusion partners include *TPR*, *C16orf72*, *IRF2BP2* and *LMNA*, while *SPECC1L*, *EML4*, *TFG*, *RBPMS* and *STRN* were reported in cases of *NTRK3* involvement [3, 8]. Rearrangement of *NUMA1* with a protein tyrosine kinase or other partners (*PDGFRB*, *ALK* and *RARA*) has been previously reported only in a handful of haematological and mesenchymal tumours [11–13], but to the best of our knowledge, *NUMA1* as an *NTRK* fusion partner has not been reported thus far in *NTRK*-rearranged uterine sarcomas or any other *NTRK*-rearranged tumours. NuMA1 (Nuclear Mitotic Apparatus protein 1) is a high molecular weight protein that plays an essential role in mitotic spindle assembly and maintenance during mitosis [14]. The detected *NUMA1::NTRK1* fusion in our patient included the N-terminal globular and coiled-coil domains of *NUMA1* and the transmembrane and kinase domains of *NTRK1* (Fig. 3). This is a consistent feature of *NTRK* fusions, where the 3′ region of an *NTRK* gene, which encodes the full kinase domain, is translocated to the 5′ region of the partner gene, which encodes an oligomerization or other protein-association domain [15]. The coiled-coil domain of the NuMA1 protein is an oligomerization domain; hence, it is predicted to promote dimerization of the fusion protein, resulting in constant activation of the tyrosine kinase domain of NTRK1.

In summary, we report a case with a novel *NUMA1::NTRK1* fusion in an *NTRK*-rearranged spindle cell sarcoma of the uterine cervix. The clinical, morphological and immunohistochemical features of this lesion are consistent with those reported in the literature, but to the best of our knowledge, this is the first description of a novel *NUMA1::NTRK1* fusion in an *NTRK*-rearranged uterine sarcoma or any other tumour type. This entity has only recently been described, but the accurate diagnosis of these rare tumours is important. The presence of CD34 and/or S100 positivity on immunohistochemistry in a uterine sarcoma of the lower uterine segment or the cervix should trigger further NTRK testing, as an *NTRK* fusion may provide targeted therapeutic options with a potential positive impact on patient survival.

## Data Availability

The datasets generated and analysed during the current study are available in the NCBI Sequence Read Archive (SRA) repository, https://www.ncbi.nlm.nih.gov/sra/PRJNA1027292.
